# Risk factors for contralateral inguinal hernia repair after unilateral inguinal hernia repair in male adult patients: analysis from a nationwide population based cohort study

**DOI:** 10.1186/s12893-017-0302-2

**Published:** 2017-11-21

**Authors:** Cheng-Hung Lee, Yu-Ting Chiu, Chi-Fu Cheng, Jin-Chia Wu, Wen-Yao Yin, Jian-Han Chen

**Affiliations:** 1 Department of General Surgery, Buddhist Dalin Tzu Chi Hospital, Chia-Yi, Taiwan; 2 Department of Colorectal Surgery, Buddhist Dalin Tzu Chi Hospital, Chia-Yi, Taiwan; 3 Department of General Surgery, E-Da Hospital, No.1, Yida Road, Jiao-su Village, Yan-chao District, Kaohsiung City, 824 Taiwan, Republic of China; 4 School of Medicine, Tzu Chi University, Hualien, Taiwan; 5 School of Medicine, I-Shou University, Kaohsiung, Taiwan

**Keywords:** Contralateral inguinal hernia repair, Contralateral exploration, Herniorrhaphy, National Health Insurance Research Database (NHIRD)

## Abstract

**Background:**

To identify the rate of and risk factors for contralateral inguinal hernia (CIH) after unilateral inguinal hernia repair in adult male patients.

**Methods:**

This retrospective cohort study identified from the Taiwan National Health Insurance Research Database (NHIRD). Information on all adult patients who underwent primary unilateral inguinal hernia repair without any other operation was collected using ICD-9 diagnostic and procedure codes. The exclusion criteria were laparoscopic hernia repair, non-primary repair, complicated hernia, other combined procedures, female and undetermined gender.

**Results:**

A total of 170,492 adult male patients were included, with a median follow-up of 87 months. The overall CIH rate was 10.5%, with a median time of 48 months to a subsequent hernia operation. The 1-year, 2-year, 3-year and 5-year-recurrent rate was 2.6, 3, 4.3, and 6.7% respectively. Further, 3.7% patients who underwent CIH repair had a complicated inguinal hernia. Multivariate analysis demonstrated that age > 45 y, direct hernia, cirrhosis (HR = 1.564), severe liver disease (HR = 1.663), prostate disease (HR = 1.178), congestive heart failure (HR = 1.138), and history of malignancy (HR = 1.116) had a significantly higher risk of CIH repair.

**Conclusions:**

Among adult male patients undergoing long-term follow-up, we identified several significant risk factors for CIH repair. If these risk factors are presented, the surgeon should inform the following risk of CIH repair to patients so that it can be repaired as soon as possible.

## Background

After unilateral inguinal hernia repair, some patients experience a contralateral inguinal hernia (CIH) and require subsequent surgical repair. Previous reports have shown that ≤30% patients develop CIH [1]. In the era of conventional hernia repair, few studies have researched the risk factors of CIH repair. Although we have identified high-risk patients, it is still difficult to determine which patients should undergo exploration of the contralateral side during the initial surgery because it may make a subsequent procedure more difficult [2, 3]. Therefore, following traditional hernia repair, surgeons typically advise patients to observe the contralateral side closely for the development of a new hernia so that a surgeon can repair it.

Unfortunately, to date, reports on adults have seldom discussed the risk factors for CIH repair. Unlike infants and children, the risk factors associated with and results of CIH repair have been widely discussed [4, 5]. Only one study used a non-Medicare claims database to analyze the risk factors for treatment of CIH and showed that an older age and prostate disease were risk factors [6].

The goal of this study was to identify the risk factors for CIH repairs in male adults based on data in the National Health Insurance Research Database (NHIRD) in Taiwan, which covers 97% of the country’s medical providers and about 99% of its citizens. Surgeons can use these data to identify high-risk patients to inform high-risk patients that they should pay attention to the possibility of a CIH so that it can be repaired as soon as possible.

## Methods

### Study sample and identification of hernia repair surgery

This study is based in part on data from NHIRD, provided by the National Health Insurance Administration, Ministry of Health and Welfare and managed by the National Health Research Institutes (registered number NHIRD-103-246). For our analysis, we extracted information on all adult patients with discharge diagnoses that included ICD-9 codes for a hernia (ICD codes 550.xx to 553.xx) combined with surgical procedure codes for unilateral inguinal hernia repair (53.00 to 53.05) [7, 8] from inpatient expenditures by admissions in NHIRD from 1996 to 2013.

**Fig. 1:**
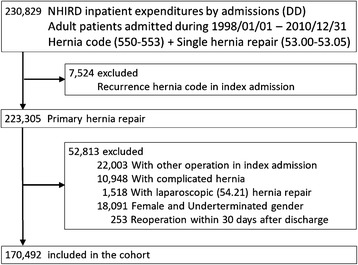
Study flow chart

Patients who underwent other operations during the same admission were excluded. Moreover, patients with a recurrent hernia (ICD coding: 550.01, 550.03, 550.11, 550.13, 550.91, and 550.93) [8], a complicated hernia (ICD-9 codes including 550.0x, 550.1x, 551.x, and 552.x [9]), women or undetermined gender were excluded. Patients discharged after undergoing laparoscopic surgery (ICD code 54.21) that indicates patients may receive laparoscopic inguinal hernia repair, were excluded. Patients who underwent reoperation within 30 days of the initial unilateral inguinal hernia repair were also excluded [10]. All patients admitted between January 1, 1998 and December 31, 2010 who fulfilled the inclusion criteria were enrolled into the analysis. All included patients were followed until their death or the end of the study period, December 31, 2013. Death was defined as withdrawal of patients from the NHI program [11]. The selection algorithm is shown in Fig. 1.

### Definition of CIH repair

The primary endpoint was CIH repair. CIH repair was defined as reoperation after more than 30 days following the initial hernia repair [10] and any of the following: 1) patients undergoing a second unilateral hernia repair, and 2) patients who underwent bilateral inguinal hernia repair after the initial unilateral inguinal hernia repair.

### Characteristics and comorbidity of patients

The patients were separated into groups based on age as follows: 18–45 years, 45–65 years, 65–80 years, and >80 years. Type of hernia (53.01, 53.03 for direct type, 53.02, 53.04 for indirect type; and 53.00, 53.05 for unspecified, respectively) and whether mesh placement occurred (53.03, 53.04 and 53.05 for repair with mesh; 53.00, 53.01, and 53.02 for repair without mesh) were also identified by ICD-9 code. During analysis, we assessed 18 independent variables as comorbidities (Appendix), including 15 different medical categories based on the Charlson comorbidity index [12]: prostate disease (ICD-9-CM code 600.x, 601.x, 602.x), which was reported as a risk factor for CIH repair [6]; obesity (ICD-9-CM code: 278.00, 278.01 [13]); and hypertension (ICD-9-CM code: 401.x-405.x [11]). Comorbidities identified by an ICD code within the NHIRD database before admission were included as comorbidities.

### Statistical analysis

We used SPSS software (IBM, Chicago, IL, USA) for the descriptive statistics and contingency tables for data analysis. Differences in CIH repair rates among age groups, gender, and comorbidities are listed in the contingency table and were compared using a chi-square test. Kaplan–Meier analysis was used to identify the percentage of patients who did not have a CIH repair in the following period. The risk of CIH repair among different covariates was evaluated using a backward stepwise Cox proportional hazards model. Variables with *P* values less than 0.2 were inserted into a Cox regression for a multivariant analysis [14]. Death before CIH repair was thought to be a competing factor and was also inserted into the Cox model for analysis. A *P* value of ≤0.05 was considered significant.

### Ethical approval and consent to participate

This study was conducted with the *Helsinki Declaration* and was fully evaluated and proved by the Institutional Review Board of Buddhist Dalin Tzu Chi Hospital approved this study (B10304006-1).

## Results

We identified 230,829 patients who underwent unilateral hernia repair between January 1, 1998 and December 31, 2010. Further, 7524 patients who underwent a repair for a recurrent hernia were excluded. We also excluded 22,003 patients who underwent other operations during the same admission: 10,948 patients underwent an operation for a complicated inguinal hernia; 1518 patients underwent a laparoscopic procedure, and 16,841 female patients and 1503 patients were of undeterminated gender. Finally, 170,942 patients were included in the study cohort. The patient selection algorithm is illustrated in Fig. 1.

### Estimation of the CIH repair rate after unilateral inguinal hernia repair

**Fig. 2:**
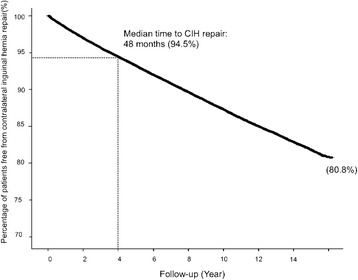
Kaplan–Meier analysis of contralateral inguinal hernia rates after primary unilateral inguinal hernia repair in male adult patients

This group had a median follow-up time of 87 months. The overall CIH repair rate was 10.5% (17,879 patients). The cumulative incidence of CIH repair, which was demonstrated by Kaplan–Meier analysis, gradually increased without abrupt fluctuations (Fig. 2). At the end of the cohort, 80.8% of the included patients did not undergo a CIH repair with a median time of 48 months from the first surgery to subsequent CIH repair. The 1-year, 2-year, 3-year and 5-year-recurrent rate was 2.6, 3, 4.3, and 6.7% respectively. There were 3.7% patients (654/17,879 patients) who underwent CIH repair for a complicated inguinal hernia.

### CIH repair rate in different clinical characteristics and comorbidities

Table 1 demonstrates the CIH repair rate in different clinical characteristics. Patients between 18 and 45 years of age had the lowest rate of subsequent CIH repair than patients in any other age group. The rate gradually increased with age and decreased in patients aged >80 years. Regarding inguinal hernia type, a direct hernia was associated with a significantly higher rate of CIH repair than an indirect hernia (direct vs. indirect = 11.8% vs. 9.4%, respectively; *P* < 0.001). Further, patients whose primary inguinal hernia was repaired without mesh had a significantly higher proportion of CIH repairs than those who were repaired with mesh (without mesh vs. with mesh = 11.4% vs. 8.0%, respectively; *P* < 0.001).

**Table 1:** Contralateral inguinal hernia (CIH) repair rate according to different clinical characteristics in 170,492 male adult patients after primary unilateral inguinal hernia repair (Overall: 10.5%; second operation: 3.7% complicated)

Clinical Characteristics	Total patients	CIH repair events	CIH Rate	*P*
Age, y				<0.001
18–45	43,075	02,541	05.9%	
45–65	59,866	07,130	11.9%	
65–80	54,503	07,134	13.1%	
> 80	13,048	01,074	08.2%	
Type of hernia				<0.001
Indirect	92,139	08,685	09.4%	
Direct	46,347	05,447	11.8%	
Unspecific	32,006	03,747	11.7%	
Mesh or not				<0.001
Without mesh	125,826	14,297	11.4%	
With mesh	044,666	03,582	08.0%	

### Risk factors for CIH in male patients

Several comorbidities showed a significant difference in subsequent CIH repair rates (Table 2), including congestive heart failure (*P* = 0.032), peripheral vascular disease (*P* = 0.023), cerebrovascular disease (*P* = 0.014), dementia (*P* = 0.001), diabetes mellitus (*P* < 0.001), renal disease (*P* < 0.001), and prostate disease (*P* < 0.001).

**Table 2:** Contralateral inguinal hernia (CIH) repair rates in different comorbidity groups in 170,492 male adult patients after primary unilateral inguinal hernia repair

Comorbidities	CIH rate		*p*
	No	Yes	
Myocardial infarction	10.5%	10.1%	=0.556
Congestive heart failure	10.5%	9.5%	=0.032^*^
Peripheral vascular disease	10.5%	8.2%	=0.023^*^
Cerebrovascular disease	10.5%	9.7%	=0.014^*^
Dementia	10.5%	6.5%	<0.001^*^
Chronic pulmonary disease	10.5%	10.5%	=0.912
Rheumatic disease	10.5%	07.8%	=0.106
Peptic ulcer disease	10.5%	10.7%	<0.521
Mild liver disease	10.5%	09.8%	0.074
Cirrhosis	10.5%	11.2%	=0.134
Severe liver disease	10.5%	11.9%	=0.055
Diabetes mellitus	10.6%	8.8%	<0.001^*^
Hemiplegia or paraplegia	10.5%	8.8%	=0.123
Renal disease	10.5%	8.1%	<0.001^*^
History of malignancy	10.5%	9.8%	=0.055
Obesity	10.5%	4.5%	=0.319
Hypertension	10.5%	10.2%	0.051
Prostate disease	10.2%	12.1%	<0.001^*^

*p < 0.05

**Fig. 3:**
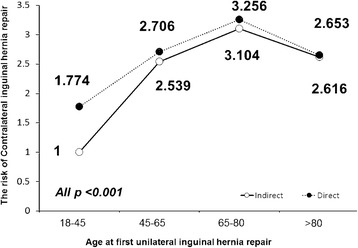
Hazard Ratio (HR) associated with Contralateral inguinal hernia (CIH) repair among inguinal hernia types in each age group

After a multivariant analysis using a Cox regression model, patients who were aged >45 years had a significantly higher risk of CIH than younger men (45-65 years: HR 2.241, 95% CI 2.140-2.347; 65-80 years: HR 2.723, 95% CI 2.594-2.859; >80 years: HR 2.306, 95% CI 2.135-2.491; all *P* < 0.001). Also, patients with a direct hernia had a higher risk of CIH repair than patients with an indirect hernia (HR 1.113, 95% CI 1.075-1.152; *P* < 0.001). Figure 3 demonstrates differences in risk among inguinal hernia types in each age group. Compared with patients aged 18–45 years with an indirect hernia, older patients had a higher CIH repair risk. Further, patients with a direct hernia had a significantly higher risk in all age groups (all *P* value <0.001).

Other factors are summarized in Table 3. Patients who underwent mesh repair for a primary unilateral inguinal hernia had a significantly lower risk of subsequent CIH repair (HR 0.887, 95% CI 0.854-0.921, *P* < 0.001). As for comorbidities, congestive heart failure (HR 1.138, *P* = 0.011), cirrhosis (HR 1.564, *P* < 0.001), severe liver disease (HR 1.663, *P* < 0.001), history of malignancy (HR 1.116, *P* = 0.004), and prostate disease (HR 1.178, *P* < 0.001) were identified as risk factors for CIH repair. Patients with diabetes had a relatively lower risk than patients without diabetes (HR 0.874, *P* < 0.001).

**Table 3:** Risk factors associated with contralateral inguinal hernia (CIH) repair rate in 170,492 male adult patients after primary unilateral inguinal hernia repair as shown by Cox regression analysis

	HR	95% CI	*P*
Characters of index operation			
Age, y			
18–45	1		
45–65	2.241	(2.140 – 2.347)	<0.001^*^
65–80	2.723	(2.594 – 2.859)	<0.001^*^
> 80	2.306	(2.135 – 2.491)	<0.001^*^
Hernia type			
Indirect type	1		
Direct type	1.113	(1.075 – 1.152)	<0.001^*^
Mesh placement			
Without mesh	1		
With mesh	0.887	(0.854–0.921)	<0.001^*^
Comorbidities			
Severe liver disease	1.663	(1.398–1.979)	<0.001^*^
Cirrhosis	1.564	(1.382–1.771)	<0.001^*^
Prostate disease	1.178	(1.129–1.230)	<0.001^*^
Congestive heart failure	1.138	(1.030–1.256)	=0.011^*^
History of malignancy	1.116	(1.035–1.203)	=0.004^*^
Diabetes	0.874	(0.820–0.932)	<0.001^*^
Renal disease	0.900	(0.793–1.022)	<=0.105
Dementia	0.840	(0.639–1.104)	<=0.221
Rheumatic disease	0.799	(0.551–1.158)	<=0.236
Hemiplegia or paraplegia	1.137	(0.898–1.441)	<=0.286
Vascular disease	0.891	(0.711–1.116)	<=0.314
Hypertension	1.015	(0.973–1.059)	<=0.485
Cerebrovascular disease	1.013	(0.941–1.090)	<=0.737
Mild liver disease	0.997	(0.917–1.085)	<=0.948

^*^Significant

## Discussion

In this study, we identified several results. First, as patients aged, the rate of CIH repair also increased. Second, several risk factors associated with CIH repair in male patients were identified, including severe liver disease (including esophageal varices bleeding, hepatic coma, portal hypertension, and other sequelae of chronic liver disease), history of cirrhosis, prostate disease, congestive heart failure, and history of malignancy. However, mesh placement and diabetes were identified as protective factors during CIH repair in male patients.

During the study, we used a Cox regression model for a multivariant analysis instead of a binary variable because we believed that the time to CIH repair was an important factor for patients. As for age as a risk factor, we demonstrated that the rate of CIH repair increases with age in male patients, which may be related to increasing muscular weakness with age. However, the risk declined in patients >80 years. In our study, we identified 59.9% patients older than 80 years who died before their CIH repair. This rate was significantly higher than in other groups (65-80 years: 37.3%; 45-65 years: 11.7%; <45 years: 5.4%; *P* < 0.001). The higher proportion of the competing event, die before CIH repair, could explain the drop in ratio and risk of CIH repair in age >80 y/o groups.

We excluded patients who may undergo a laparoscopic hernia repair (LIHR). Although there was no strong evidence to prove that LIHR significantly decreased the CIH rate, as compared with a traditional hernia repair, the reported CIH rate after LIHR of about 1–5% was lower than that after traditional inguinal hernia repair of 8–22% [15–19]. Moreover, as we previously mentioned, contralateral exploration during LIHR has been reported to be an easy and safe procedure. Some previously asymptomatic, unrecognized synchronous hernias may have been repaired during laparoscopy and thus could have been recorded as a bilateral inguinal hernia repair in the NHI database, which may influence the overall rate and make the risk factors we identified have more confounders. Therefore, we excluded this issue from our study cohort.

Previously, in the era when tension-free hernia repair was not popular, sequential bilateral inguinal hernia repair may have been performed with a 1-week interval with general anesthesia or at least a 48-h interval with local anesthesia for recovery and mobilization [20, 21]. Moreover, in our clinical experience, some surgeons would repair bilateral hernias separately because of patient comorbidities. Under the NHI system of Taiwan, patients who are readmitted within 14 days would be considered as having incomplete treatment or complications from a previous admission; thus, the payment would be less. If surgeons choose to repair bilateral inguinal hernias, patients would be admitted >14 days after a previous discharge date. Moreover, Saleh et al. indicated that reoperation within 30 days was thought to be an unreliable variable because a particular reoperation may not be related to the initial surgery [10]. Thus, we excluded patients who underwent reoperation within 30 days.

Based on our results, the direct hernia is a risk factor for CIH repair in men. It may indicate greater abdominal wall weakness if patients develop a direct hernia and explain why patients with a direct hernia have a higher risk of CIH. As for prostate disease, severe liver disease, and cirrhosis, we thought these conditions had a higher risk because of increased abdominal pressure and chronic straining [6]. Previously, one Korean journal indicated that the incidence of long-term recurrence and contralateral metachronous hernia repair after unilateral inguinal hernia repair in liver cirrhosis patients does not differ from that of patients without cirrhosis [22]. However, this previously mentioned study involved fewer cases (129 patients with liver cirrhosis) than our study. We believe that our result provides a more accurate prediction of the risk of CIH repair.

In recent years, mesh placement during hernia repair was thought to be the gold standard for inguinal hernia repair. A recurrence rate of 0.2%–25% was demonstrated, and recurrence decreased by about 50%–75% after inguinal hernia repair, as compared with conventional non-mesh methods [23]. Even after 10 years of follow-up, mesh repair is still superior to non-mesh repair because of significantly lower recurrence risks (1% vs. 17%, respectively; *P* = 0.005) [24]. Further, no data in literature have demonstrated that mesh repair would increase or decrease the risk of CIH repair. Our result showed mesh repair could lower the risk of CIH repair. It might be related to the foreign body sensation after mesh repair. It was reported that 20% patients might have foreign body sensation, 3.7% hyperthesia and 19% hypothesia of the inguinal area after mesh repair [25]. This may remind the patient continuously and influenced patients’ daily activity, reduce the chance of lifting heavy materials and cause the decrease of contralateral inguinal hernia repair.

As for other comorbidities including a history of malignancy and congestive heart failure served as risk factors, and diabetes served as a protective factor, to our knowledge, no previous journal articles mentioned these factors. For the history of malignancy and congestive heart failure, chronic illness and relatively poor nutrition status may play a role in the development of CIH. In the other hand, patients with diabetes are often taught to take care of their limbs to prevent unhealed wound. This may retrain their activity amount or intensity. Probably, the risk of contralateral inguinal hernia decreased. However, the definitive mechanism is still unclear. These identified factors may provide surgeons a guideline to perform contralateral exploration during laparoscopic hernia repair for unilateral inguinal hernia or to inform high-risk patients the possibility of developing a contralateral inguinal hernia. However, mechanisms regarding the protective effect of mesh and diabetes treatment in contralateral inguinal hernia repair are still unclear. Further study is necessitated to clarify the mechanism.

### Limitations

This study had some limitations. First, for our analysis, we used only inpatient expenditures by admissions. There were no records on repairs performed in outpatient departments, repairs in other countries, or self-paid repair during the follow-up period. Some patients who underwent an operation in the outpatient department would not be included in the cohort. However, we believed that considering patients who were admitted for hernia repair would accurately reflect the rate of significant CIH. Consequently, we believed that this rate could help surgeons identify patients who would need contralateral exploration during laparoscopic unilateral inguinal hernia repair or surgeons advise those high-risk patients who should pay attention to symptoms of CIH after traditional inguinal hernia repair.

Second, we could not delve into the details of each physician’s notes to determine the type of mesh placed, mesh fixation methods, or any other factors such as changes in technique and experience of the surgeon, the severity of cirrhosis, or stage of malignancies that were not recorded in the database or identified by ICD-9. Thus, we could not conduct additional subgroup evaluations on these topics. However, we used NHIRD, which covers 97% of medical providers in Taiwan and about 99% of its citizens and records all medical behaviors in the country. Thus, we could detect contralateral inguinal hernia repair once a patient was admitted in Taiwan. We thought that such a long followed-up period could detect almost all contralateral inguinal hernia repair in Taiwan and offer a comprehensive and reliable data source for the present study.

Third, the risk of miscoding exists. In the NHI database, upon admission, diagnosis and procedures performed were recorded according to ICD-9. However, surgeons in Taiwan have always used a different coding system, Health Insurance Surgical Orders from the Taiwan NHI payment system, which directly relates to the revenue earned by surgeons. However, as it was directly related to hospital income, professional coders provided mostly these records in this database that were based on the entire admission course. Moreover, an official comparison table is offered by the National Health Insurance Administration Ministry of Health and Welfare to help increase the preciseness of these different codes. Moreover, we believed that the likelihood of miscoding of the surgical procedures was limited.

## Conclusion

For patients with a median follow-up of 87 months, 10.5% of the patients needed to undergo CIH repair with a median of 48 months from the first surgery to subsequent CIH repair. The 1-year, 2-year, 3-year and 5-year-recurrent rate was 2.6, 3, 4.3, and 6.7% respectively. Moreover, 3.7% of these patients experienced a complicated hernia. We identified several significant risk factors for CIH repair following traditional unilateral hernia repair including age >45 years, direct hernia, and comorbidities including cirrhosis, severe liver disease, prostate disease, congestive heart failure, and history of malignancy. Patients who were repaired with mesh had a relatively lower risk. If these risk factors are present, surgeons should inform patients that they should pay attention to the possibility of a CIH, so it can be repaired as soon as possible.

## Appendix

**Table 4:** Translation of comorbidities into ICD-9-CM Codes

Diagnostic Category	ICD-9-CM Codes	Description
Myocardial infarction	410-410.9	Acute myocardial infarction
	412	Old myocardial infarction
Congestive heart failure	428-428.9	Heart failure
Peripheral vascular disease	443.9	Peripheral vascular disease, including intermittent claudication
	441-441.9	Aortic aneurysm
	785.4	Gangrene
	V43.4	Blood vessel replaced by prosthesis
	Procedure 38.48	Resection and replacement of lower limb arteries
Cerebrovascular disease	430-438	Cerebrovascular disease
Dementia	290-290.9	Senile and presenile dementias
Chronic pulmonary disease	490-496	Chronic obstructive pulmonary disease
	500-505	Pneumoconioses
	506.4	Chronic respiratory conditions due to fumes and vapors
Rheumatologic disease	710.0	Systemic lupus erythematosus
	710.1	Systemic sclerosis
	710.4	polymyositis
	714.0-714.2	Adult rheumatoid arthritis
	714.81	Rheumatoid lung
	725	Polymyalgia rheumatica
Peptic ulcer disease	531-534.9	Gastric, duodenal and gastrojejunal ulcers
	531.4-531.7	Chronic forms of peptic ulcer disease (subset of above listing)
	532.4-532.7	
	533.4-533.7	
	534.4-534.7	
Mild liver disease	571.4-571.49	Chronic hepatitis
	070.22, 070.23, 070.32, 070.33	Chronic hepatitis B
	070.44,070.54,	Chronic hepatitis C
	070.6*, 070.9*	Unspecified hepatitis
Cirrhosis	571.2	Alcoholic cirrhosis
	571.5	Cirrhosis without mention of alcohol
	571.6	Biliary cirrhosis
Severe liver disease	572.2-572.8	Hepatic coma, portal hypertension, other sequelae of chronic liver disease
Diabetes	250-250.3	Diabetes with or without acute metabolic disturbances
	250.7	Diabetes with peripheral circulatory disorders
	250.4-250.6	Diabetes with renal, ophthalmic, or neurological manifestations
Hemiplegia or paraplegia	344.1	Paraplegia
	342-342.9	Hemiplegia
Renal disease	582-582.9	Chronic glomerulonephritis
	583-583.7	Nephritis and nephropathy
	585	Chronic renal failure
	586	Renal failure, unspecified
	588-588.9	Disorders resulting from impaired renal function
History of malignancy	140-172.9	Malignant neoplasms
	174-195.8	Malignant neoplasms
	200-208.9	Leukemia and lymphoma
	196-199.1	Secondary malignant neoplasm of lymph nodes and other organs
Hypertension	401-405.9	Hypertension
Prostate disease	600-602.9	Prostate disease
Obesity	278.00, 278.01	Obesity

## Abbreviations

CI: Confidence interval
CIH: Contralateral inguinal hernia
HR: Hazard ratio
LIHR: laparoscopic hernia repair
NHI: Taiwan National Health Insurance
NHIRD: National Health Insurance Research Database
